# Urban planning in Swiss cities has been slow to think about climate change: why and what to do?

**DOI:** 10.1007/s13412-022-00767-9

**Published:** 2022-06-18

**Authors:** Gilles Desthieux, Florent Joerin

**Affiliations:** 1 Institut du Paysage, d’Architecture, de La Construction Et du Territoire (inPACT) Haute Ecole du Paysage, d’ingénierie Et d’architecture de Genève (HEPIA), Rue de la Prairie 4, CH-1202 Geneva, Switzerland; 2 Institut d’ingénierie du Territoire (INSIT) Haute Ecole d’Ingénierie Et de Gestion du Canton de Vaud (HEIG-VD), Route de Cheseaux 1, CH-1401 Yverdon-les-Bains, Switzerland

**Keywords:** Climate governance, Land use planning, Swiss climate policy, Stakeholder involvement

## Abstract

Recent years have been marked by a strong popular and political mobilization around climate change. However, to what extent does this mobilization lead to reduce greenhouse gas emissions or the vulnerability of our society to the effects of climate change? This question is at the heart of the research presented, which sought to identify the barriers and levers to the integration of climate issues into urban planning of Swiss cities. The literature review first situates the integration of climate change in Swiss cities in relation to the evolution of practices at the international level. It emerged that Swiss cities have generally been late in integrating climate issues into their public policies. Practices still focus strongly on energy policies aimed at reducing greenhouse gas emissions, but adaptation measures in urban planning are poorly implemented. In order to better understand the reasons for this slow and late integration of climate change into urban planning of Swiss cities, a survey was conducted among more than 200 professionals. It showed that the evolution of practices is generally driven by “pioneering” actors who are strongly mobilized by personal values and who use specialized and scientific sources of information. Finally, two focus groups with representative professionals were organized in order to deepen the barriers and levers observed and to formulate sound recommendations for integrating the climate issue into urban planning. Two lines of action emerged: prioritization (strengthening legal frameworks and organizational structures) and support (training and involvement of climate experts at all stages of urban planning).

## Introduction

Events related to climate change are increasingly evident and have further accelerated in 2021. At the same time, awareness at all levels of society is increasing, thanks in particular to the 6th IPCC report (July 2021), which further highlights the state of emergency. The challenge is to move from awareness to concrete action, and to overcome the many institutional obstacles that were highlighted during the recent summit COP26 in Glasgow.

Switzerland is particularly exposed to climate change. If the temperature of the earth has globally increased of about + 1 °C since the preindustrial area, in Switzerland the increase is of about + 2 °C (FOEN 2020), mainly due to the particular mountainous context. However, until the early 2010’s Switzerland did not really develop any climate change policy, particularly in terms of adaptation.

The 2013 CO_2_ Act is the basis for Switzerland’s current climate policy. It sets a greenhouse gas emissions reduction target of at least 20% by 2020 compared to 1990 levels; this target must be achieved through measures taken in Switzerland. According to Federal Office for the Environment - FOEN (FOEN 2022), total equivalent CO2 emissions effectively decreased of 20% between 1990 and 2020, therefore achieving the target. Transportation represents the main source of CO_2_ emissions: 32% at national level, and even more 39%, if international flights are considered. Emitted CO_2_ by inhabitant at national level is only of 5.5 tCO_2_/inhabitant. But this ratio increases until 14.2 tCO_2_/inhabitant, according to www.globalcarbonatlas.ch, if we consider the energy consumption of imported goods, which makes Switzerland the 36th emitter in the world (132nd by surface area). The CO_2_ Act was revised in September 2020 at the Swiss parliament. A major element under discussion is the achievement of carbon neutrality by 2050, involving, however, a significant part of compensation abroad.

Until recently, Swiss energy and climate policy has mainly focused on mitigation aspect of climate change. A study conducted in 2011 (Dupuis and Knoepfel 2011), based on lexical analysis of policy makers’ discourses, demonstrated that adaptation was still not perceived as an important dimension of climate change policy: policy makers did not see links between adaptation, the Swiss economy and the energy supply. However, awareness on adaptations issues has raised during the last years. Mountain areas being more and more exposed to natural hazard events, and cities to summer overheating. In this context, the Swiss government adopted in 2012 (FOEN 2012) a general strategy on climate adaptation followed in 2014 by the Action plan 2014–2019 (FOEN 2014), and finally guidelines for support Cantons in the elaboration of their Climate Plans (FOEN 2015). In order to implement the action plan, the Federal Office for the Environment—FOEN launched a program to support pilot projects at regional and local levels: 31 projects supported in the first stage (2013–2017) (FOEN 2017), 50 projects supported in the second stage (2018–2022). Cantons (states in the Swiss federal system) and local communities are now taking initiatives to develop regional and local climate actions on both aspect mitigation and adaptation.

The recent strengthening of Swiss climate governance is partly the result of the population’s awareness toward climate emergency that took to the streets in Switzerland, as elsewhere in the world, during 2019. On several occasions, youths and schoolchildren organized strikes for climate, and media widely covered climate issues. This certainly contributed to the historic score of the green party in the last federal election in October 2019, becoming the fourth party at national level.

Regarding land use planning, it can be noted that planners are also raising awareness of the importance to take into account (adaptation) climate issues. For example, the 2019 national workshop of the Swiss Federation of Urban Planners (FSU) was dedicated to this topic (FSU 2019). However, until recently, Swiss cities, like most cities in the world, had not been very active on climate change issues (Lehmann et al. 2015). Yet, the municipal level is particularly decisive in transposing the different framework conditions for climate policy to the operational level (Scanu and Cloutier 2015; McClure and Baker 2018; Harker et al. 2017).

To sum up, it can be pointed out that in Switzerland, the governance of climate issues, particularly on adaptation, has only become a reality at the national level since around 2010 and is only beginning to be implemented at the local level. The starting point of this research is therefore a questioning of the reasons for a certain delay in the implementation of concrete measures on the Swiss urban territories. In the specific Swiss context, the general question is similar to the one addressed by Lehman et al. 2015: “What are relevant barriers to effective (adaptation) actions in cities and what are possible opportunities for progress?”.

This research considers both mitigation and adaptation of CC, but it particularly focuses on the adaptation topic, which is central in land use planning for reducing the effect of heat waves in urban areas or the exposure to natural hazard. The rest of the article is organized in four main parts. First, it presents a brief state of the art (Section 2) of research about the relationship between climate change and territory, followed by a presentation of the applied research method (Section 3). The next part (Section 4) describes the results of the research, which consists of three sections: first, a portrait of the Swiss institutional and organizational mechanisms (actual and potential) that are implemented to integrate climate change into land-use planning practices, second the results of a survey of spatial planning actors in French-speaking Switzerland that has been conducted that was conducted in order to better understand what facilitates or hinders the integration of climate change in professional practice, and third, a set of operational recommendations for the consideration of climate change mitigation/adaptation issues in urban projects resulting from group discussions. The article ends with a conclusion and discussion (Section 5).

## State of the art

Considering the recent literature, this section analyzes what are the key climate change issues for urban planning in European and North American cities and how climate change is taken into account in land use planning, whether adaptation or mitigation is considered.

### Adaptation and mitigation in urban planning

The definitions of adaptation and mitigation are already well given in the literature. Mitigation is “a human intervention to reduce the source or enhance the sinks of greenhouse gases” (IPCC 2014, p. 4), while adaptation “primarily aims at moderating the adverse effects of unavoided climate change through a wide range of actions that are targeted at the vulnerable system” (Füssel and Klein 2006).

To sum up, Bertrand and Richard (2015) give a very helpful synoptic view of both concepts. Climate policy was first mainly based on mitigation in order to limit GHC emissions. Mitigation mainly involves technological and economic sciences and a more top-down oriented governance due the central role of authority for regulation and subsidies as observed by Scanu and Cloutier (2015). Its impacts are relatively easy to measure (GHC reduction, innovation) and the win–win for different actors is often obvious. Adaptation is more multi-disciplinary oriented; its impacts and benefits are sometimes less measurable and uncertain. Its mode of governance is more bottom-up oriented based on engagement and collaboration of many local actors (Scanu and Cloutier 2015). The priority consideration of both mitigation and adaptation varies according to the cultural and institutional context as shown in the comparative study made by Scanu and Cloutier (2015) between Quebec City (Canada) and Genoa (Italy).

An assessment of local climate plans from 885 cities in the EU-28 (Reckien et al. 2018) showed that only 26% of those plans deal with adaptation, and 17% with both mitigation and adaptation. Similarly, a worldwide study on 401 municipalities (Araos et al. 2016) reported that only 18% of them set up planning toward adaptation policy. Therefore, cities still mainly focus on mitigation in order to seek carbon neutrality in the context of climate emergency (Salvia et al. 2021). If the need of human adaptation measures to climate action seems always more evident through the vast and fast-growing on this topic, Berrang-Ford et al. (2021) as well as Olazabal and De Gopegui (2021) state that adaptation is overall not effectively implemented. Local impacts and effects of adaptation are limited; capacity to reduce vulnerability and risk is lacking.

While mitigation seems easier to achieve at local levels, because in synergy with energy constraints and orientations, adaptation requires more specific appropriation work, based on existing institutional mechanisms. The question is how to make territories resilient to climate change effects (Berdoulay and Soubeyran 2014). In the literature, many examples of adaptation measures can be found:

- Green infrastructure shape adaptation preferences among residents in Rotterdam (Derkzen et al. 2017), opportunities and gaps for urban green infrastructures planning in Europe (Davies and Lafortezza 2017).
- Cartography of heat islands in Graz based on GIS indicators (Reischl et al. 2018)
- Land use planning related to sea level rise and extreme weather events (McClure and Baker 2018)
- Summer thermal comfort related to urban form and compactness in Berlin (Straka and Sodoudi 2019).
- Cloutier et al. (2014) organized in Quebec a design workshop with professionals who identified 18 sectoral planning measures of 4 types: (1) coating materials (color, texture, albedo, etc.), (2) urban form (height of buildings, street orientation, etc.), (3) natural cover and (4) building architecture.

Those examples show that adaptation is mainly carried out into sectoral activities. But adaptation has not yet been seen as really transversal program driving land use planning. According to Cloutier et al. (2014), adaptation can become a mean of innovation for planning process, which does not involve to initiate new actions or processes but to improve and orient the existing ones toward climate change issues. It can even become a new gateway to sustainability science in land use planning (Bertrand and Richard 2015).

In this perspective, new approaches should be proposed for land use planning instruments, dealing with municipal land use revision, new neighborhoods developments, open spaces planning in existing neighborhoods. This involves working on a global and transversal framework, and not only sectoral, and to investigate how far such integrated approaches could be transposed into the regulations at different levels. Propositions in this matter will be made through the article using the Swiss context.

### Levers and barriers of taking into account climate change in urban governance

One of the main challenges is to modify land use planning practices in order to systematically consider climate change issues. Different authors account for levers and barriers of taking into account climate change in planning processes. United Nations Intergovernmental Panel on Climate Change (IPCC) distinguishes between physical and ecological limits, technological limits, financial barriers, informational and cognitive barriers, and social and cultural barriers (IPCC 2007). Important works and surveys were carried out to identify barriers and levers. For instance, Olazabal and De Gopegui (2021) studied planning in 59 cities worldwide, and Simonet and Leseur (2019) conducted 75 semi-structured interviews among 75 actors in 10 French cities.

The following table (Table 1) gives a selection of frequent occurrences of barriers and levers classified into three main topics: knowledge sharing, financial resources, organization and regulation:

**Table 1:** Main barriers and levers for climate governance as referenced in the literature

Barriers		Levers	
Items	Authors	Items	Authors
Knowledge sharing			
Lack of actual data on implementation and effectiveness	Olazabal and De Gopegui (2021),	Set up adaptation monitoring, evaluation, reporting and learning in order to track how policy processes connect to adaptation success	Olazabal and De Gopegui (2021),
Low engagement of local actors: misperception of cause-and-effect linkages of climate change impacts, lack of awareness and anticipation	Cloutier et al. (2014), Lemann et al. (2015), Simonet and Leseur (2019)	Reach out to a diversity of stakeholders, increase common knowledge and understanding of the issues, change in individual perception	Cloutier et al. (2014), Burton and Mustelin (2013), Simonet and Leseur (2019)
Lack of understanding among actors, deficit in efficient communication between researchers, policy makers and the public	Cloutier et al. (2014), Reischl et al. (2018) Eisenack and Stecker (2012	Use of external supports providing expertise, technical and cognitive resources (boundary organizations)	Henstra (2016), Richard (2014), Scanu and Cloutier (2015)
Lack of consideration of local knowledge and need of vulnerable groups	Olazabal and De Gopegui (2021)	Use of social networks for improving information sharing	Cunningham et al. (2016)
Financial resources			
Lack of financial resources, reduction of national subsidies	Henstra (2016) Eisenack and Stecker (2012), Simonet and Leseur (2019)	Financial incentives, subsidies, tax expenditures Green economic growth, innovation	Henstra (2016) Scanu and Cloutier (2015)
Organization and regulation			
Planning decisions not enough targeted to climate issues, difficulty of choosing the best strategy	Cloutier et al. (2014), Mees et al. (2018)	Systematic introduction of climate change issues (adaptation) in public policies: habitat and urbanism, naturel hazard and water management, health, agriculture, biodiversity, etc	Richard (2015)
Lack of integration of adaptation plans in current institutional, regulatory and financial framework,	Olazabal and De Gopegui (2021), Scanu and Cloutier (2015), Moser and Ekstrom’s (2010)	Reorganization of public institutions for implementing climate policies in order to guarantee sustainable adaptation action in the long-term and change in individual perceptions Identification of alternative authorities and rationale (boundary organizations) and action margin in existing capacities (land use planning)	Olazabal and De Gopegui (2021), Simonet and Leseur (2019), McClure and Baker (2018)
National/upper level regulation not compatible with climate change issues	(Harker et al., 2017), (McClure and Baker 2018)	Elaboration of Local Climate Action Plan	Harker et al., (2017), Richard (2015)

Faced with the observation of these barriers and levers, the question is how urban governance could evolve to facilitate the consideration of climate change in the development of cities (Bulkeley and Betsill 2013).

### Toward an urban governance for climate

In the specific field of adaptation, Henstra (2016) proposes a framework to structure climate governance in general. It classifies policy instruments, to which governments have access, in four main axes:

- Nodality (information): Use of information dissemination, knowledge generation and knowledge mobilization to inform responses
- Authority: Use of the legitimate power of the state to permit, prohibit or command action
- Treasure: Use of public funds to (1) produce and maintain public goods and services that contribute to improve climate governance; (2) confer benefits to induce new behavior; or (3) impose costs to discourage behavior that undermines new behavior
- Organization: Use of government resources and personnel to implement policy objectives

Combining those instruments in complementary ways is the key to maximize the likelihood that objectives will be realized.

Information and its different forms and the adaptation of the regulation frameworks (related to authority axis) are the necessary first steps that precede the action, whereas institutional resources (related to organization axis), with the support of financial means and incentives, enable to pass from the knowledge to the concrete action.

Concerning Nodality, bottom-up approaches based on knowledge sharing among actors are central in order to overcome institutional silos that split climate change knowledge, measures and responses into isolated and ineffective policies. They include participatory approaches through workshops and forums like in Quebec City (Cloutier et al. 2014), social networks for engaging local communities in climate adaptation policy like in Australia (Cunningham et al. 2016), questionnaires and interviews gather perceptions of decision makers or residents on climate impacts in cities like in Graz, Austria (Reischl et al. 2018) or in Rotterdam, Netherlands (Derkzen et al. 2017), and boundary organizations.

Boundary organization is particularly helpful platform, which aims facilitating interaction or mediation between science and policy (Gustafsson and Lidskog 2018). In the field of climate policy, it makes available to the stakeholders impartial, technical and cognitive knowledge and resources for guidance on how to respond to the threat of climate change (Henstra 2016; Richard 2015; McClure and Baker 2018). This kind of organization acts as motivators by pushing local governments to take action and by translating experts’ jargon into understandable credible form for decision makers and the civil society. The Ouranos nonprofit consortium based in Quebec is often cited. Created in 2002, it has the mandate to provide advices to its governmental, academic and private partners (Huard et al. 2014).

About the 3 others axes Authority, Treasure and Organization, Harker et al. (2017), as many other authors (Mees et al. 2018; Scanu and Cloutier 2015; McClure and Baker 2018), emphasize the role of cities and local governments in climate policy implementation. However, the local actions on climate should take place in the perspective of multi-level governance that considers articulations with the upper levels: international, national and regional (Lehmann et al. 2015; Harker et al. 2017). The use of suitable governance and organizational instruments should therefore consider whether states are centralized (unitary systems) or federal. In the latter, local governments have more autonomy in developing their own system of regulation, taxation and subsidies and thus their own climate policy. In some particular contexts, centralized governments may constitute institutional barriers when political parties and regulations are unfriendly with climate change issues. This has been the case for example in Italy (Scanu and Cloutier 2015), New Zealand (Harker et al. 2017) and Queensland in Australia (McClure and Baker 2018). Those authors also show how local governments and actors can deal with this kind of barrier with the support of boundary organizations, and some action margin through developing specific regulations (land use planning related to natural hazards). In this context of lack of regulatory framework, the involvement of individual motivations and beliefs of professionals is crucial for engaging local actions (McClure and Baker 2018). In comparison with the mentioned above conservative federal systems, Swiss national authorities promote an ambitious climate policy (see Section 3.1) and supports local implementation actions. Even if cantons (states) and municipalities have a relative autonomy, such a federal impulsion enables to boost local actions.

The framework proposed of Henstra is one among others proposed in the literature. For instance, Lubell and Morrison (2021) developed a framework for institutional navigation they applied to climate change adaptation based on four elements: knowledge (similar to the Nodality axis of Henstra), relationships, strategies, decisions and implementation. This framework addresses both collective (institutional) and individual goals. This latter distinction is important, because, as we will see through the survey in Section 4.2, professionals of urban planning intervene and decide not only in the name of the organizations they represent, but also and often according to their personal values. Therefore, interpersonal relationships are crucial to reach agreement on climate change actions.

## Methodology

The methodological approach is structured into three main stages which is introduced here in this section. The results of each stage are then respectively presented in Sections. 4.1, 4.2 and 4.3.

The first stage deals with how far climate change issues have been considered or not in the Swiss context, particularly about adaptation. Concretely, the content of the climate policy instruments and tools was analyzed from regional to local levels using the framework introduced below in Section 3.1.

Secondly, a questionnaire was collected from about 200 professionals in the field of land use planning in order to investigate how far they take into account climate issues in their practice, what are the barriers and levers for such considerations (Section 3.2).

Finally, two focus groups with a representative panel of professionals were organized in order to deepen observed barriers and levers and formulate sound recommendations for integrating the climate issue into land use policy practices (Section 3.3).

### Analysis of governance instruments for climate issues in Switzerland

In order to draw up a critical overview of different practices of integration of climate issues in the field of spatial planning in Switzerland, at different scales, the working method consisted in collecting a set of documents representative of the actions carried out and instruments elaborated in the field of climate governance in Switzerland by different organizations (public, academic, private). These documents were then analyzed and compared according to a common analysis grid.

The instruments considered (presented in the table in Appendix 1) are classified in four categories that cover the areas of action in climate governance:

1. Local projects and initiatives,
2. Regulation and climate action plan,
3. Land-use planning instruments,
4. Labels and evaluation processes.

Several programs and projects referenced in connection with these instruments are part of the pilot projects supported by the Confederation and the Federal Office for the Environment—FOEN (see Section 1). The aim of this selection is not to be exhaustive by referencing the whole set of the known instruments, but to provide a representative overview of the initiatives in Switzerland up to 2019 when the study was conducted.

The analysis framework is structured under 14 rubrics as presented in Table 2. For each rubric, it is indicated to which axis of climate governance it mainly belongs (information, authority, treasury, organization), as presented in Section 2.3.

**Table 2:** Analysis grid structured in 14 rubrics that was applied to the Swiss instruments

Rubric	Analyzed content/questions	Policy instrument type (Henstra 2016)
1. Abstract	What it is about in broad outline, articles/chapters/part explicitly related to climate change	N.A
2. Mitigation vs, Adaptation	Does the analyzed document address these two pillars of climate change in a balanced way, or is one of the two preponderant?	Information
3. Objectives of the instrument	What are the objectives, the intentions of the instrument that is the subject of the document under review? Is the issue of climate change central (explicit) or secondary (implicit)? The objectives are often also multiple (multi-functionality): do the measures have effects on areas other than climate change? Vice versa, do measures that do not directly address climate change have indirect impacts?	Information
4. Topics addressed by the instrument	What topics does the action address and to what extent those topics a more or less strongly concerned? Level of coordination (between thematic areas): are the instruments and actions concerned implemented in an integrated and coordinated (multi-sectoral) manner, or in a sectoral, non-integrated (juxtaposed) or even ad hoc manner?	Information
5. Regulation actors	List of concerned actors and the nature of their involvement (In charge, Associated, Concerned)	Organization
6. Scale of action	What scale(s) in particular does the area of action cover? How territorialized are the instruments (general principles vs. spatial and geographic impacts)?	Organization
7. Funding	Are financial resources made available to implement the measures/actions? What is the distribution between the private and public sectors?	Treasury
8. Degree of intervention	To what extent the instruments are binding for concerned people? Are they informative (diagnosis), organizational (e.g., willingness of authorities but without regulatory translation), regulatory or incentive (subsidy or taxation) in their nature?	Authority
9. Format of the medium and degree of concreteness	What is the format of the instrument? What level of knowledge? Is it a global diagnosis study report? Or does the instrument materialize into an action/measure plan?	Information
10. Degree of territorial coordination	How coordinated are the actions/instruments with the measures (plans, regulations) established in the same portion of the territory (internal, or intra-territorial coordination) and with the higher and lower territorial levels (external, or extra-territorial coordination)?	Authority
11. Temporality of deployment	Is the temporality of the instruments defined? If so, what are the main steps?	Authority
12. Monitoring of implementation strategy	Are qualitative or quantitative targets set for climate change objectives? According to which monitoring system (indicators)?	Information
13. Degree of technicality	To what extent do the suggested instruments involve more or less sophisticated/specific tools?	Information
14. Overall appreciation of the instruments	How original and innovative are the suggested instruments in terms of: goals/objectives pursued, topics addressed, actors involved, instruments used (in an integrated way), steering and monitoring?	Organization

### Survey among the actors of regional planning

In a second phase, we wished to explore the reasons for the slow and partial integration of climate issues in the practice of urban planning in Switzerland. For this reason, we carried out a survey among (French-speaking) actors in this professional field.

The online survey was distributed and made available from January 30 to March 8, 2019. The target audience consisted of professional actors in the field of spatial planning in municipal and cantonal administrations, private offices (mainly urban planning, but also architecture and transport), associations and academics in the French-speaking part of Switzerland.

The survey was distributed directly by email to more than 500 people (professionals involved in the field of land use planning, selected from the authors’ professional network) and 231 responses were obtained. An invitation was also sent to various professional mailing lists (associations of urban planners) for which the number of subscribers is not known. In addition, those who received the invitation message were also encouraged to pass it on to others concerned. Therefore, this method of distribution does not allow for the measurement of a response rate.

The survey consisted of 49 questions, structured into four main parts:

1. Professional practices: specifies the field of activity, describes how climate change is taken into account, and the factors that are favorable (levers) or unfavorable (barriers) to considering it.
2. Climate Change Information: describes the knowledge of climate change and its effects and the uses of the available information.
3. Individual practices: explores the consideration of climate change in individual practices (mobility, housing, food, etc.).
4. Personal profile: professional affiliation, age, education, political beliefs.

The average response time was 17 min.

As pointed out by Lehmann et al. 2015, it is important to go beyond the identification of barriers and levers to also study their interactions. Thus, the analysis of the survey results is largely based on statistical tests aimed at highlighting interdependencies between the different responses collected.

### Focus groups

In order to follow up on the results of the survey and to open up paths for action, we organized a process in two focus groups bringing together actors in the field of urban planning. These aimed to progressively define priority action sheets. The participants were selected from the authors’ professional network so as to cover the following topics: the realization of climate plans for two large cities (300,000 to 500,000 inhabitants), the urban planning of a medium-sized city (23,000 inhabitants), the urban development of an agglomeration, within a large real estate development company, or in urban planning consultancies or specialized in energy and climate issues. A dozen people participated in each of the two focus groups. The majority of participants were able to attend both sessions, but about 1/3 of them were only available for one of them.

The first focus group was held on October 8, 2019 in Geneva. It began with the presentation of the research project, followed by the presentation of the state of the art (Section 2), the analysis of the Swiss context (Section 4.1) and finally the presentation of the results of the survey (Section 4.2). Afterward, sequences of work in pairs and then jointly allowed the participants, including the research team (both authors), to formulate their point of view on the blocking factors (barriers) to the integration of climate change into urban planning. Finally, the participants were asked to identify, in pairs and then jointly, a first set of courses of action and levers to address the identified obstacles.

The second focus group, which took place on October 17, 2019 in Lausanne, started with a reminder of the results obtained in the first session. Afterward, participants were asked to select one or two courses of action (called “Measure”) in order to describe in more detail their content and implementation process. Appendix 2 provides an example of a form completed by one of the groups on one of the identified action lines.

Each focus group was designed to produce diversified and directly exploitable results (factsheet, schemes). It was therefore not necessary to transcribe the discussions between participants; however, during each focus group a notetaker was present to produce a summary note which was returned to the participants for validation.

## Results

### Climate change instruments and tools in Switzerland

The analysis of the documentation collected and processed according to the grid presented above (Table 2) provides useful lessons on the implementation of territorial and climate governance in Switzerland at the local level. The main results are presented below, using the headings of the analysis grid.

#### Mitigation vs. Adaptation (see rubric 2 in Table 2)

Until recently, climate policy in Switzerland was closely linked to energy policy and mainly focused on the mitigation of GHG emissions. This focus on mitigation is probably due to the direct and logical link between efficient use of renewable energies and emission reductions. Thus, more than 400 municipalities (in 2019), supported by the Confederation, have committed themselves to establish an exemplary energy policy through the Energy City label (Swiss contribution to the European Energy Award program).

Recently, mitigation and adaptation seem to be more closely linked in Swiss climate policy. One example is the new criteria catalog of the “Cité de l'énergie” label, which integrates requirements relating to quality of life and the fight against global warming in public spaces and buildings, as well as adaptation measures accordingly.

Land use planning is gradually becoming a major axis of action in relation to climate change, both on the mitigation side (reducing CO2 emissions in terms of mobility, construction and rational use of energy) and on the adaptation side (urban development in relation to natural risks, adapted urban forms). The successive Climate Plans of the Canton of Geneva (2015, 2017, 2021) is one of the first significant illustrations of how climate issues can be taken into account in a spatial planning instrument. This evolution at the cantonal level (region) is to be underlined but it is still only slowly reflected at the lower levels.

#### Objectives and topics of the instruments (see rubrics 3 and 4 in Table 2)

Overall, the climate issue is still rarely mentioned explicitly in the objectives of the urban plans. It appears rather implicitly and indirectly through the various disciplines concerned with climate and in particular adaptation: green and public spaces, urban climate, health, energy and mobility. This interdisciplinary framework highlights apparent conflicts, in particular between adaptation and urban density. Indeed, high urban densities, which are encouraged by urban planning policies in Switzerland, constitute particular challenges in terms of heat islands or vulnerability to climatic hazards. This delicate link between urban planning and climate policy (Xu et al. 2019, Biesbroek et al. 2009) is well illustrated in Zurich where a wind map shows that light winds and breezes, especially at night from nearby forests and mountains, have difficulty circulating in dense urban areas, thus accentuating night-time warming (Stoiber 2019).

Thus, planners are becoming increasingly aware of the importance of integrating climate measures into the construction and densification process. For example, the Climate plan of Geneva highlighted the benefits of climate action in all three areas of sustainable development (echoing the link observed by Bertrand and Richard 2015, between sustainability science and adaptation). At the social level, the measures envisaged can contribute to improving air quality, fighting sedentary lifestyles or strengthening food security. From the environmental point of view, they contribute to preventing floods, strengthening biodiversity, increasing soil fertility or preserving natural resources. As for the economic effects, these measures contribute to supporting the local economy, developing new skills or reducing material damage.

#### Regulatory actors (see rubric 5 in Table 2)

The coordination and arbitration of public policies, in close partnership with economic and civil society actors, appears to be a central component in the implementation of climate governance measures. In the Mitteland region (canton of Lucerne), a pilot project consists of establishing a region-wide climate change adaptation strategy and encouraging political actors to take into account the possible consequences of climate change when making future regional development decisions and projects. This involves conducting a broad discussion based on the available information, developing a catalog of measures and providing support to municipalities for the implementation of the strategy.

#### Degree of territorial coordination (see rubric 10 in Table 2)

The interdisciplinary and multisector approach required for climate governance confronts the actors involved with the issue of coordination between planning instruments. The institutional frameworks analyzed indicate three forms of response. Some, such as the cantons, develop integrated and autonomous climate plans, others, more often at the communal level, include climate-related issues in all existing planning instruments, and still others develop the climate topic in sectoral plans (environmental impact study, energy concept, mobility study, etc.).

#### Scale of action (see rubric 6 in Table 2)

The instruments analyzed show that actions are gradually being implemented at all spatial scales: the Climate Plan in Geneva and the Mitteland at the regional level (with measures creating favorable framework conditions for operational achievements), local initiatives in Bern (study on the role of trees in urban climate), Lausanne (green roofs), Zurich (mapping of microclimatic phenomena), Sion (climate-related planning of public spaces) and very recently an acceleration in the development of the climate plans at the municipal level.

#### Format and degree of concretization (see rubric 9 in Table 2)

Climate issues are mainly addressed in reports, studies or diagnoses. They thus take the form of directives, general and indicative principles, and guides to good practice: Lausanne concerning green roofs, in Sion for the planning of spaces, in Zurich concerning the urban forms of constructions to be favored in relation to the climate. These principles are still rarely translated into concrete measures.

#### Degree of intervention (see rubric 8 in Table 2)

Our study shows that, in Switzerland, there are still generally few or no legal obligations to consider climate change, nor are there any sustainable subsidy mechanisms. At the municipal and neighborhood levels, the topic of climate is rarely mentioned in spatial planning guidelines. However, the situation is changing: the Climate Plan of the Canton of Geneva recommends, for example, adapting the Geneva legalization to further limit fossil fuels in new or converted buildings, or adapting regulations and standards relating to (grey) energy in construction, and changing the guidelines for municipal development plans, environmental impact studies and energy concepts to better take climate issues into account. From now on, any local development plan (municipality) in this Canton will have to refer explicitly to the measures of the Climate Plan and specify the contributions of the municipalities in this area. At the level of cities and municipalities, Sion is the best-known example in Switzerland of integrating climate change adaptation measures into local planning and building regulations and plans (the City of Sion being one of the most affected in Switzerland by heat islands). In a more sectoral manner, the City of Lausanne imposes the greening of roofs in urban planning regulations as compensation for the loss of green surfaces on the ground.

#### Financing (see rubric 7 in Table 2)

Although the issue of financing measures is central to their implementation, the documents analyzed make little mention of this aspect. On the mitigation side, it is relatively easy to calculate the cost of the measures and also to evaluate the returns on investment, which generally involves the renovation of buildings and the development of renewable energy. The adaptation component is much less clear-cut, both in terms of the cost of the measures (is it already integrated into sectoral or additional measures) and in terms of the induced benefits, which are often indirect and difficult to perceive. Furthermore, as mentioned by Lehmann et al. 2015, costs of adaptation arise in the short term while benefits often in the long term. The climate plan for the Zürich region describes examples of synergies between different measures that make it possible to reduce the overall cost of their implementation. Another area of savings consists in developing preventive measures rather than emergency measures in response to climatic hazards. For example, according to the latter document, it is much cheaper to make people at risk aware of the dangers of heat waves than to have to treat them afterward, or, for example, it is cheaper to identify and regulate areas at risk of flooding or other natural hazards than to have to deal with the numerous damages caused by high exposure to hazards.

Very often, the implementation of actions requires public financial assistance (Lehmann et al. 2015). This is the case at the national level through pilot projects supported by the Federal Office for the Environment—FOEN or at the local level, with the cities of Sion and Lausanne providing financial support for initiatives. An active and financial involvement of public actors thus still seems essential to take concrete account of climate change on the (Swiss) territory.

#### Summary

Table 3 summaries the main findings from the Swiss instruments in regard with the structure presented in Table 2.

**Table 3:** Main findings from the Swiss climate change instruments

Rubric	Outcomes
1. Abstract	-
2. Mitigation vs, Adaptation	Climate policy is still closely linked to energy policy and mainly focused on the mitigation of GHG emissions until recently Land use planning is becoming a major axis of action in relation to climate change, both on the mitigation side
3. Objectives of the instrument	Climate issue is still rarely explicitly mentioned in the objectives of the urban plans Awareness is raising about integrating climate measures into the urban planning process and their multisector benefits
4. Topics addressed by the instrument	
5. Regulation actors	Importance of coordination and arbitration of public policies, in close partnership with economic and civil society actors for the implementation of climate governance measures
6. Scale of action	Instruments are not limited to general intentions but progressively being implemented at local scales through emerging climate municipal plans
7. Funding	Financial aspects are generally considered for mitigation measures, but more rarely for adaptation measures: indeed, the benefits related to risk management are revealed in the long term
8. Degree of intervention	Still few legal obligations address climate change. But the situation is rapidly evolving through the climate commitments made at all levels
9. Format of the medium and degree of concreteness	Climate issues are mainly addressed in reports, studies or diagnoses and until recently rarely translated into concrete measures
10. Degree of territorial coordination	Climate policy is expected to become a bridge between planning instruments, but in practice it is still often developed in a sectoral manner
11. Temporality of deployment	Temporality mainly considers progress toward zero carbon. Monitoring is implemented to track carbon emission reduction but still few about adaptation progress
12. Monitoring of implementation strategy	
13. Degree of technicality	This issue is not explicitly carried out in the analyzed instruments, but it logically addresses technical measures to reduce carbon emissions
14. Overall appreciation of the instruments	The study revealed some pilots project that are original and innovative, to be spread out and generalized

On the basis of these analyses, the hypothesis that Switzerland has presented until recently a slow integration of climate change into territorial governance instruments and a weak concrete implementation of the recommendations can be confirmed. This slow and weak implementation can be seen in regard to climate urgency that was already acknowledged in the early 2010s in Switzerland, and in comparison with some other European and North American cities as presented in Section 2. The validation of this hypothesis is mainly revealed by the following findings:

### Survey of levers and barriers to changing land use planning practices

#### Introduction and general profile respondents

The online survey (see 3.2) received 231 responses. The respondents’ fields of training are mainly in engineering, territorial sciences (geography, urban planning, development) and natural sciences, and finally management (Table 3). The respondents’ activities are mainly related to issues related to the management of public services, spatial and urban planning, environmental protection, transportation or mobility (Table 4).

**Table 4:** Profile of respondents on field of training

Fields of training	Count	%
Engineering	66	29%
Territorial sciences (geography, urbanism, planning, etc.)	54	23%
Natural, biological and earth sciences	50	22%
Management and administration	46	20%
Other humanities and social sciences (political science, economics, sociology, etc.)	31	13%
Architectures	17	7%
Law studies	7	3%
Physical and mathematical sciences	7	3%
Others	32	14%

Slightly more than half of the respondents (52%) declared political values close to environmentalist parties, whereas these parties received in Switzerland (in September 2019) just under 20% of the citizen votes. It will therefore be a question of interpreting the results of this survey bearing in mind that respondents often show a prior sensitivity to climate change issues.

While the analysis of the literature and territorial governance instruments shows that climate change issues are still only partially integrated, a strong majority of respondents (84%) say they take climate change into account in their professional activities, implicitly or explicitly (Table 5). Of these, the majority of respondents (56%) say they take climate change into account implicitly, which means that they do so through pre-existing measures, that were not made specifically to address issues of climate change. As an illustration, one might find in this category a person working in public transport development. On the other hand, 38% of them say that they do so explicitly, which means by setting objectives to be achieved or actions to be carried out directly related to climate change.

**Table 5:** Profile of respondents on field of activities

Activity focus	Count	**%**
Public service management or administration	107	46%
Spatial planning or urbanism	97	42%
Biodiversity and environmental protection	65	28%
Transport or mobility	60	26%
Energy production and distribution	36	16%
Climatic changes	33	14%
Others	25	11%
Teaching and research	24	10%
Agricultural sector	23	10%
Economic development	17	7%
Natural risk and hazards	14	6%
Public health	9	4%
Digital sector	9	4%

This first result could be seen as a discrepancy between, on the one hand, the respondents’ perception of the integration of climate change into their activities and, on the other hand, the results of our analysis of the literature and institutional instruments in Switzerland (Section 4.1), which only very partially reflect the systematic consideration of climate change. However, these responses should be interpreted with caution since, as previously mentioned, the sample of respondents presents a profile that is particularly sensitive to environmental issues. It may also be the result of a time lag between a relatively recent change in practice that was not yet very present when the documents or instruments analyzed (see 4.1) were written or developed.

#### Levier and Barriers

Two questions in the survey addressed the levers or barriers to integrating climate change issues into respondents’ professional practice (Table 6). The responses obtained place the commitment of the institution (public or private) as the most important lever (almost 50% of respondents). Then we find, with relatively equal importance, regulatory change, access to subsidies. Then, but for 1/5 of the respondents, we note that a particular climatic event can also constitute a trigger (Table 7).

**Table 6:** Degree of consideration of climate change in professional activity (several possible choices) and main results

In your professional activity, is climate change taken into account?	
Results	**%**
Explicitly, through the setting of objectives to be achieved or actions to be taken	38,1
Implicitly, through already existing measures (risk management, planning, project sizing, etc.)	55,8
There is no consideration of climate change in my professional activities	18,6

**Table 7:** Results on barriers and levers from the survey

Levers	**%**
Commitment of a department, a team, an employee	46,3
Political commitment from the elected representatives of your territory	46,3
Public subsidies	24,5
Private initiative project(s)	23,9
Change in the legal or regulatory framework	23,4
Significant climatic events in your area	20,2
Collaboration with a third-party specialist	18,6
Consequences for activities in your region	18,6
Others	9,6
Barriers	**%**
Other higher priority issues	48,5
Lack of financial resources (to work on)	43,7
The cost of the required actions (to implement)	35,9
Lack of human resources or skills	34,2
Limited or unknown consequences of CC effects	24,7
Lack of interest from your managers, colleagues, funders	23,8
The limited impact your activities could have on the climate change issues…	19,9
Others	11,3
Climate change controversies	6,5

With regard to barriers, the one most often chosen was the existence of issues associated with a higher priority. This result is linked to the lack of financial resources, which is almost as often chosen. It can also be noted that lack of skills is identified as a barrier by more than 1/3 of respondents. Among those least often selected, it is interesting to note the feeling of being powerless (The limited impact your activities…) or the controversies on climate change. Finally, 11% of respondents chose the “other” category, among which some mentioned that work routine is sometimes also a barrier.

#### Interdependences analysis between answers

In order to try to understand what leads respondents, who are actors in land-use planning, to integrate climate change, explicitly or implicitly, into their professional activities, independence tests (Chi-square) were carried out to highlight the absence or presence of associations between the answers collected.

**Fig. 1:**
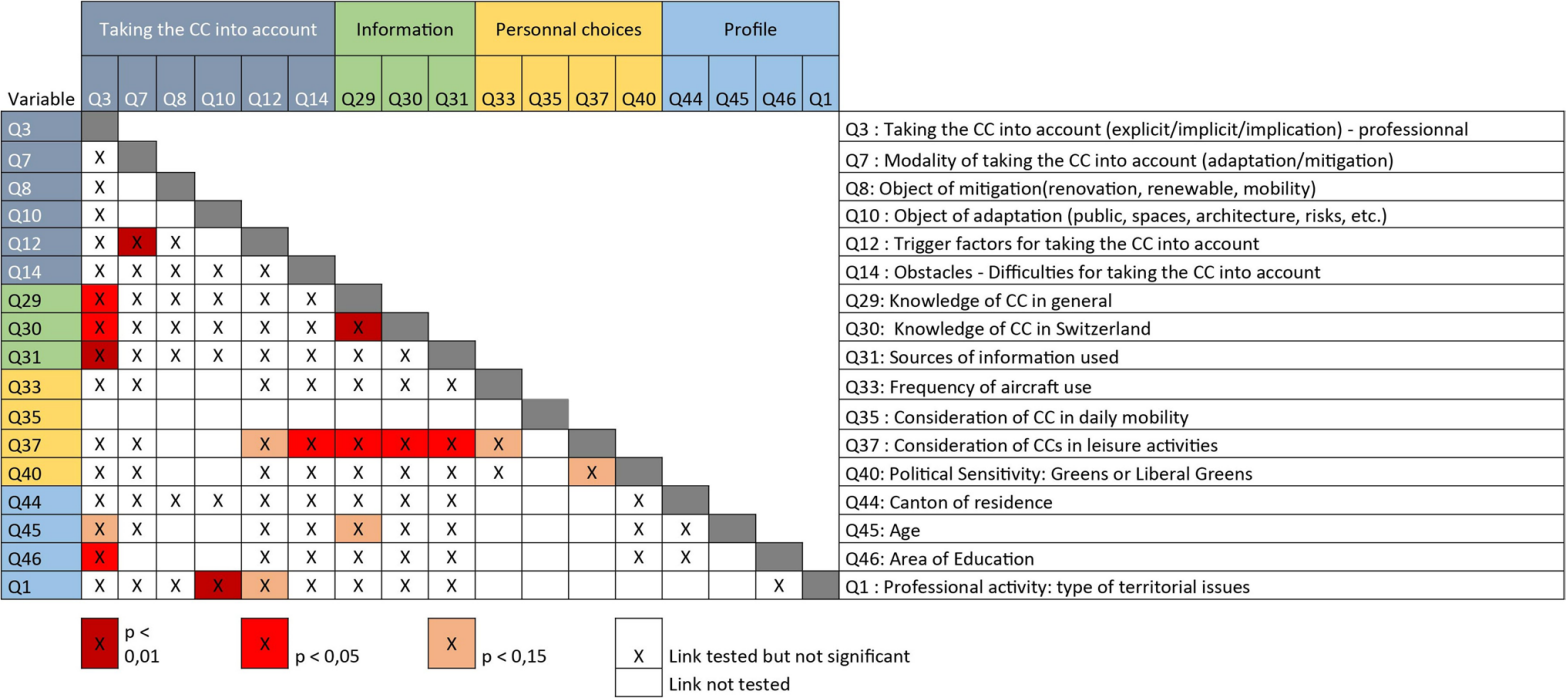
Table of dependency relationships between the answers to the questions asked in the survey

Figure 1 presents all the independence tests carried out. The questions are organized into four main blocks exploring: 1) how CCs are integrated into professional practices, 2) the level of knowledge and the use of available information, 3) how CCs are integrated into private practices, 4) personal characteristics. Two questions can be observed which present several significant dependency relationships: question 3, already mentioned, which deals with the consideration of climate change and question 37 on the consideration of climate change in private leisure activities.

The analysis presented here focuses on the statistically significant interdependencies between the consideration of climate change in professional activities (Q3) and variables in three main themes: knowledge and access to information (Q29 to Q31), age and education (Q45, Q46) and personal choices (Q35).

The presence of a cross indicates that the independence test has been completed. The use of colors expresses the degree of significance of the test (*p* < 1%, *p* < 5%, *p* < 15%). For example, a p value = 1% means that there is a 99% probability that the variables are dependent. The white boxes (with a cross) are not significant.

#### Profile of respondents toward integration of climate change into professional practices

At the date of the survey, February 2019, which is important to remember because the political context of climate change is changing rapidly, the analysis of the results seems to highlight a group of committed actors who take climate change explicitly into account in their professional activities. This group of actors, which we describe as *pioneers*, is distinguished by two main factors. First, they also integrate climate change issue in their personal choices. In particular, an interdependence is observed between taking climate change into account in professional practices and personal mobility choices. Second, pioneers are making more than others an active use of available sources of information on climate change and its issues. These sources of information consist of conferences, scientific publications, documents produced by public administrations or training courses. It also seems that this group of pioneering actors is more often present in the lower age brackets (< 36 years) and in technical training, in engineering or architecture, for example. It also seems that this group of pioneering actors is more often present in the lower age categories (< 36 years) and in technical training, in engineering or architecture, for example.

At the same time, we observe a group of actors, less involved, who take climate change into account through pre-existing measures and who mainly inform themselves through the press. These people are generally older, inform themselves through the press and have less technical training, which suggests that climate change issues are still perceived as having little to do with the social sciences or humanities.

The table below (Table 8) summarizes the factors that are favorable and unfavorable to the integration of climate change into the respondents’ professional practices.

**Table 8:** Summary of the favorable and unfavorable factors for taking climate change into account

Respondent characteristics	Values associated with: “taking climate change into account”	Values associated with: “not taking climate change into account”
Considers CCs for personal mobility	Yes	No
Training	Technical training: engineering, architecture, natural sciences	Land use planning, human sciences, law
Source of information used	Specialized sources and training on CC	Press
Age	< 36 years	> 50 years

This result confirms that the information availability (related to the Nodality axis as presented in Section 2.3) is a determining factor in the change of professional practices (Lehmann et al. 2015). However, it also shows that availability of information is not sufficient in itself since only the most motivated actors seem to know, use and appreciate the available sources of information (Henstra 2016).

It is also noteworthy that these pioneers cite the importance of subsidies as a lever for action slightly more often than others and, even more strongly, they point to the lack of interest from their managers, colleagues, funders or clients as a hindrance (Q37 and Q14). It can thus be assumed that they are faced with (passive) resistance from their professional context, which could be interpreted as an enrolment issue. They would encounter difficulties in convincing their colleagues or superiors of the importance of better taking climate change into account.

Finally, in general, professional contexts or fields of activity do not seem to play a determining role. In other words, the implementation of concrete and targeted actions on climate change would rather be driven by the personal commitments of individuals to these issues in their private lives. The absence of significant dependencies between the variables describing professional contexts and the consideration of climate change allows us to hypothesize a lack of institutional guidelines or objectives that could be endorsed by the actors of these institutions, regardless of their personal convictions or values. However, if an evolution of these institutions through the commitment of pioneering actors seems a natural progression, a more effective consideration of climate change in general practices of land use planning would require their rapid transposition into coherent and more widely applied institutional strategies. These findings on the importance of pioneering actors are also consistent with the conclusions of the literature on the influence of the specific characteristics of actors (Lehmann et al. 2015) and their individual motivations in the context of a lack of regulatory framework (McClure and Baker 2018) in relation with the Authority axis.

### Focus group: toward courses of action

#### Outcomes from the focus groups

**Fig. 2:**
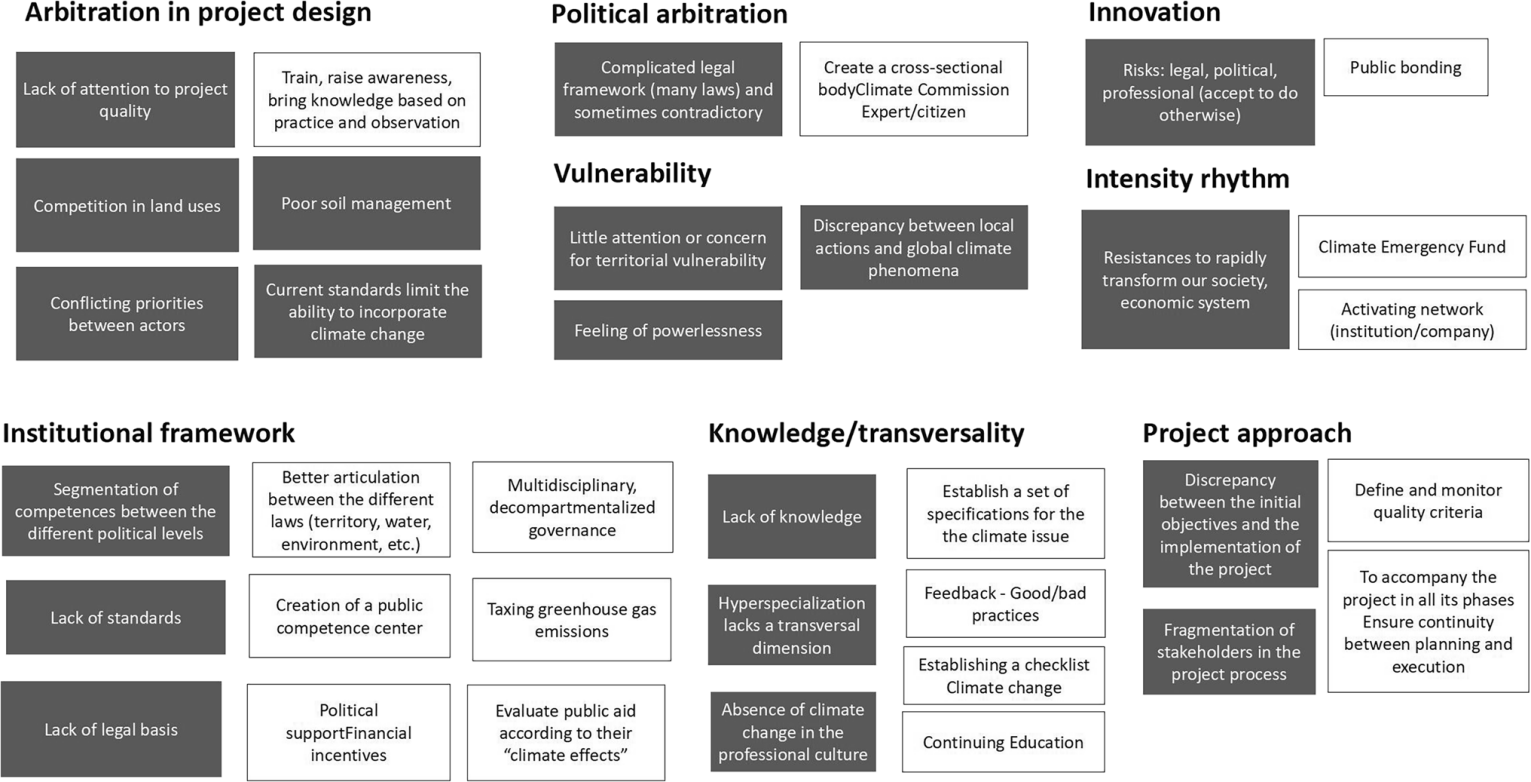
Main levers and barriers identified during the first focus group section. Black boxes: Barriers / White boxes: Levers

The first focus group allowed participants to share their views on what facilitates (levers) or hinders (barriers) the integration of climate change into the professional practice of land use and urban planning. Figure 2 presents a synthesis of these discussions. Barriers and levers are grouped into main topics. This result gives a wide variety of opinions and proposals. However, issues of lack of information and legal framework, and the need for arbitration between conflicting policies are the most prevalent.

**Fig. 3:**
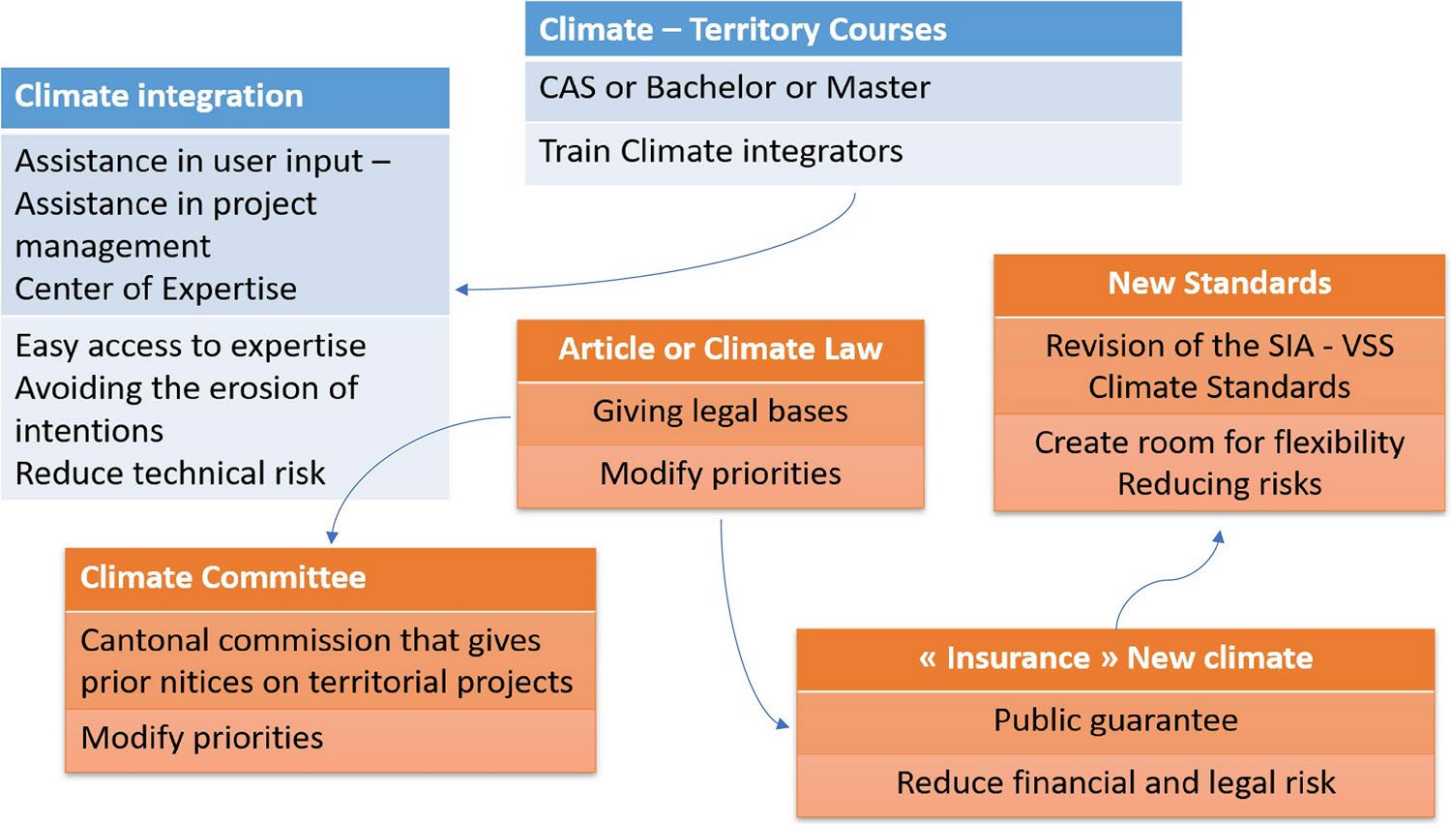
Priorities for action and connections that were discussed during the second focus group meeting. SIA and VSS refer to Swiss norms (SIA: Swiss society of engineers and architects, VSS: Swiss Association of Road and Transport Professionals)

Based on the identification of barriers and levers during the first focus group, the very rich exchanges that took place during the second focus group enabled to highlight six major priorities for action as presented in Fig. 3.

The arrows conceptually illustrate the interrelations between the priorities for action. Indeed, they are not independent, the achievement of one can facilitate the achievement of the other, but they can be carried out in parallel. Their presentation can be made by considering two structuring axes: 1) the need to reinforce the weight of climate change among the other issues and topics in urban planning decisions 2) the need to support the actors in the integration of climate issues in their professional practices. The proposed actions can also be easily associated with the four categories proposed by Henstra (2016): Nodality, Authority, Treasure and Organization.

#### Giving more weight to climate change

The first action described is of the Authority type. In order to rebalance interests in favor of climate change, the participants considered it desirable to *modify the legal bases* of urban or territorial projects by introducing a constitutional (cantonal) article or a (cantonal) law requiring the consideration of climate change in urban planning plans and projects. Indeed, the participants particularly mentioned that once the already existing legal constraints (that are not concerning climate change) have been respected, project designers have neither the resources (time and money), nor the room for maneuver to still integrate climate issues. However, they also discussed the paradox that adding legal constraint concerning climate change would make the legislative framework even more complex. So, some of them argued for giving more latitude to the interpretation of legal constraints and the balancing of interests, rather than adding new legal constraints further limiting the possibility of finding solutions specifically adapted to the local context.

In the same vein, participants pointed out that the *professional standards and codes* that apply to urban projects are not always or rarely compatible with a relevant and effective consideration of climate change. For example, parking standards, which require a minimum parking rate per dwelling based on distance from the urban center, can be an obstacle to the implementation of a strong soft mobility strategy or the fight against the sealing of public spaces. In addition, as with the findings on legal constraints, the application of the many existing professional standards also considerably reduces the room for flexibility needed to integrate climate change into territorial projects. The participants therefore suggested that a rapid review of standards and codes from a climate change perspective (construction sector, urban planning) be undertaken to verify their consistency and open up areas for action.

Another proposed course of action to reinforce the priority of climate issues is the creation of a *cantonal climate commission* whose mission would be to formulate advance notice of urban projects (neighborhood plan and building permits). This is an organization-type action. Similar to existing thematic commissions, such as the one present in some Swiss cantons on natural hazards, this commission would bring together representatives of different services (health, safety, environment, nature, etc.). This commission could, on one hand, guarantee that climate issues are taken into account and, on the other hand, be the place where interests are weighed between the different standards or constraints that apply to the project concerned. However, one limitation of this action is obviously to introduce an additional stage in the authorization procedure for urban projects.

Finally, related to Treasury axis, participants considered that the low priority given to climate change is also due to the presence of technological, financial and legal risk. Indeed, the integration of climate change into urban projects often requires departing from usual and standardized (see above) practices. Thus, project leaders are afraid of exposing themselves to technical faults that may involve financial charges or legal proceedings as well as delivery delays (involving penalties). These risks may arise, for example, from the use of new construction techniques or materials (recycled concrete, wood, etc.) or from innovative, less controlled and routine practices in urban planning and the development of public spaces (choice of suitable coverings, for example), for which experience and know-how are less consistent. It would therefore be in the interest to reduce these risks by setting up a system of *public guarantee*. In other words, it would be a matter of making available to project leaders a kind of public insurance offering coverage on the implementation of technological innovations. This could contribute, from their point of view, to getting professionals out of the routines of usual businesses (construction sector in particular).

#### Supporting the integration of climate issues

In order to accompany the actors of the regional planning toward a modification of the professional practices in favor of the climate stakes, the participants considered that it would be necessary to train *“climate integrators”* intervening in the urban project approaches. Indeed, the results of the survey showed that access to information on climate change and its relationship to the territory is a determining factor in the evolution of practices. Thus, there is a real challenge in facilitating its appropriation by the presence of relay actors.

Through CAS (Certificate of Advanced Studies), Bachelor’s or Master’s degrees, the aim would be to *train* urban planners, developers, planners or builders specializing in climate change. These people could offer specific skills related to the climate and its consequences, as well as transversal skills related to the project approach. However, the participants were aware that this is a medium-term perspective, requiring nearly ten years between the definition of educational programs and the entry into the market of climate integrators.

Another form of accompaniment could be the one offered by a *center of competences* (boundary organization). Like the Ouranos consortium in Quebec (see Section 2.3), this center of expertise could produce targeted studies, make recommendations, promote exemplary projects, but also answer questions or provide more general support to project leaders in integrating climate change.

By facilitating access to *urban climate expertise*, it would be possible to reduce the observed erosion of intentions and motivations, which are often strong in the early stages of projects, but become residual at the implementation stage. Moreover, the positive effects of facilitated access to the necessary skills are not limited to the design and implementation of urban projects, but could also contribute to achieve better environmental performance in the operational phases. The participants thus evoked the interest in developing skills in the area of climate issues not only in the form of assistance to the project owner, i.e., a person who supports the project manager, but also in the form of a user manager, this time supporting the users of the project, once it has been carried out. Indeed, it is often observed that the expected performance in terms of energy consumption, for example, is not finally achieved due to inadequate user behavior.

All the actions proposed concerning the support of the integration of climate issues are in the Nodality type as proposed by Henstra (2016), aiming at mobilizing, reinforcing and sharing knowledge.

## Discussion and conclusion

This article presents, step by step, the investigation of a general question: how to help Swiss urban planning actors to better integrate climate change into their professional practice?

In addition to a review of scientific literature, data collection was carried out through Swiss case studies, a survey of professional circles and focus groups.

These approaches did not produce an exhaustive portrait of climate practice in Switzerland, which was not the objective; however, they did bring out very instructive insights with a view to strengthening and generalizing climate governance in Switzerland.

This research shows that actors in the field of spatial planning in Switzerland have been slow to realize the need to modify their practices to face climate change. This observation and the reasons that we have put forward to understand them are in many respects similar to elements already observed in other contexts. In Switzerland, as elsewhere, it is relatively recent that urban instruments or projects explicitly address climate change issues. Moreover, many of the projects that do so are still pilot projects subsidized by public institutions.

However, the analysis of the Swiss situation also shows, in the case of a federal-type system, a good articulation between the federal, cantonal and local levels according to the principles of subsidiarity. Thus, an ambitious climate policy will at the federal level has been undertaken to give impetus and encourage the implementation of actions at the cantonal and local (municipal) levels. This top-down approach could preserve Switzerland from the problems observed in other federal contexts (Harker et al. 2017 in New Zealand and McClure and Baker 2018 in Australia) where local action is often hampered by a lack of ambition at the higher level.

The study also showed that federal initiatives are slowly (and recently) being taken up at the lower political scales (cantons), which are the most decisive in spatial planning practice. However, given the lack of clear and sustained guidelines from these cantonal or municipal institutions, the evolution of professional practice is strongly linked to the personal motivations of individual actors. There is thus certainly a step to be taken to institutionalize and perpetuate, in spatial planning practices, the importance of meeting the challenges posed to urban and territorial systems by climate change.

Although the courses of action that were identified in focus groups are partly specific to the Swiss context, they nevertheless easily fit into the four categories proposed by Henstra (2016), showing that the proposed conceptual framework is adapted to structure climate governance in various contexts. This result thus underlines the importance of pursuing a strategy that combines these different fields of action.

It seems important to us to return to the central role of information. Indeed, our results show that, on the one hand, actors who are aware of the issue are more informed and that, on the other hand, actors who are better informed act more in favor of taking climate change into account. In other words, feeling informed facilitates action, but access to information is still restricted to previously motivated actors. We could thus assume the existence of a vicious circle that restricts the group of actors who are ready to modify their professional practices: one should be motivated to gather information and one should be informed to be motivated. From our point of view, the key to this dynamic is therefore certainly easier access to (widely) available information. Fortunately, several public institutions are diversifying their modes of communication (videos, discussion groups, participatory approaches, social networks, etc.).

It should be noted that throughout this research, but also and especially since the distribution of the questionnaire, awareness of the climate emergency has accelerated in Switzerland through numerous street demonstrations (climate strike) and the confirmation of a “green wave” in the last federal elections (October 2019). The current COVID-19 pandemic will certainly also have had an impact on motivations, either by reinforcing them in favor of climate (since these events demonstrated the possibility of acting in emergency and the benefits of a society slowing down its activities), or by leaving aside the climate theme in the face of the importance given to other health and socioeconomic emergencies. The year 2021 has also been decisive in the awareness of the climate emergency through the climate hazards that have multiplied and the alarmist tone of the latest IPCC report.

This context, where climate phenomena, awareness and policy responses are evolving very rapidly, raises the question of the *internal validity* of the instruments presented in the article: analysis framework for climate policies in Switzerland, questionnaires and surveys, focus group framework. The results presented in Section 4 for each instrument are representative of the current state of awareness and opinions during the research project period (2018–2019). We assume that if the instruments were being applied presently, the results would have been different. For example, the number of climate plans in cantons and cities has increased since 2020. The questionnaire distributed 6 months later would probably have put more emphasis on personal convictions and the role of information, which has also greatly increased in recent months. The perception of priority actions (see Fig. 2) resulting from the focus group sessions would also probably be different now. While the validity period of the results is short, we believe that the proposed structure of the instruments remains valid over a longer term. As a perspective, it would be interesting to apply these same instruments on a regular basis to reflect the evolution of climate policy implementation, professional practices and opinions. Concerning the *external validity*, which addressed the *generability* of the process, the question is particularly how far the sample of the involved people can be extended. Indeed, both interviewees (survey) and participants of the focus group were selected among professionals involved in climate-related fields in order to target climate policy levers. But since it is acknowledged that climate is now a concern for society as a whole and for every individual, we believe that the proposed approach and tools could be applied to the entire professional spectrum and to the public in general, with minor adaptations in the formulation of the questions. The focus groups could also be used to facilitate public forums in the neighborhoods. This would allow us to broaden the range of responses and proposals.

Finally, if the picture of the situation described at the time of this study has indeed changed significantly in recent years and if, moreover, the Swiss situation presents certain specificities (economic and political) which limit the comparison with other countries, we are nevertheless convinced that the barriers and levers identified in this study are transposable to other situations where changes in practices must be implemented in a short time. In Switzerland, as elsewhere, the integration of climate policies into spatial planning is underway, but many steps remain to be taken and these may present difficulties similar to those identified in this article.

## Appendix 1

The table presents the detailed view of the instruments analyzed in the Swiss context. For each instrument, it is indicated whether it addresses Adaptation (A), Mitigation (M) or both.

**Table:**

Tool	Description	Organism	Scale	A	M
1.Local projects and initiatives					
Urban green infrastructures & climate (Blaser et al. 2017)	Study of the link between climate change and trees in cities. Development of methods and concepts for sustainable urban tree management. Pilot project supported by FOEN (phase 1)	City of Bern	Municipality	x	
Green roofs Lausanne (www.lausanne.ch/toitures-vegetalisees)	The City owns some 3,000 roofs and is making every effort to promote the greening of its own roofs. It also encourages homeowners and professionals to welcome nature on their roofs	City of Lausanne	Municipality	x	
AcclimataSion (Ville de Sion 2017)	The City of Sion carried out urban development projects that give priority to vegetation and the water cycle, developed a guide for professionals, and guidelines for construction and development projects. Pilot project supported by the FOEN (phase 1)	City of Sion	Municipality	x	
2.Regulation and climate action plan					
Energy law Genève	Requirements for the construction and renovation of buildings with regard to renewable energy, efficiency; energy planning of neighborhoods and municipalities	State of Geneva	State (canton)		x
Climate plan Genève (Genève 2018)	Strategic, transversal and operational instrument structured in two parts. Component 1: inventory and strategies of the climate policy via 6 axes. Part II: measurement sheets for each axis	State of Geneva	State (canton)	x	x
Climate plan Zurich (Bättig et al. 2013)	Studies carried out at different scales integrating the analysis of the effects of climate change in different fields and the proposal of priority actions and measures. Elaboration of thematic maps of heat islands and winds contributing to cooling	State and city of ZH	State, region and city	x	x
Climate plan Mitteland (Kholer and Kraus 2016)	Analysis of the effects of climate change on a lowland and medium mountain region still little affected, adoption of an adaptation strategy, mobilizing the network of key actors. Pilot project supported by FOEN (phase 1)	Region	Region	x	x
3.Land use planning instruments (Geneva)					
Directive on master municipal land use plan (Genève 2016)	Cantonal directive concerning the updating of communal master plans, with a chapter on the climatic aspect but not very detailed. Climate is mainly dealt with through the communal energy plan and the fight against emissions	State of Geneva	Municipality	(x)	x
Local urban planning	Analysis of a few development neighborhood plans adopted in 2017 and 2018 and related documents. Climate addressed only with reference to emissions of pollutants, GHGs and O_3_ depleting substances, but explicit adaptation measures	State, builders	Neighborhood		x
4.Labels and evaluation processes					
European energy award (https://tool.european-energy-award.org)	The “Cité de l'énergie” label rewards municipalities that actively pursue a sustainable energy and climate policy. The latest version of the Management Tool contains more climate-related criteria, particularly with regard to adaptation	OFEN	Municipality	x	x
Site 2000 watts (https://www.2000watt.swiss/en/english.html)	This label rewards neighborhoods (planning, transformation, operation) that conform to the principles of a 2000-W society. Criteria mainly concern attenuation; others relate to the quality of public spaces guaranteeing thermal comfort	OFEN	Neighborhood	x	(x)

## Appendix 2

The figure below gives an example of a measure described by one of the groups during the second focus group meeting.
